# Molecular switch from MYC to MYCN expression in MYC protein negative Burkitt lymphoma cases

**DOI:** 10.1038/s41408-019-0252-2

**Published:** 2019-11-20

**Authors:** Lucia Mundo, Maria Raffaella Ambrosio, Francesco Raimondi, Leonardo Del Porro, Raffaella Guazzo, Virginia Mancini, Massimo Granai, Bruno Jim Rocca, Cristina Lopez, Susanne Bens, Noel Onyango, Joshua Nyagol, Nicholas Abinya, Mohsen Navari, Isaac Ndede, Kirkita Patel, Pier Paolo Piccaluga, Roshanak Bob, Maria Margherita de Santi, Robert B. Russell, Stefano Lazzi, Reiner Siebert, Harald Stein, Lorenzo Leoncini

**Affiliations:** 1 Department of Medical Biotechnology, University of Siena, Siena, Italy; 2 Cell Networks, Bioquant, University of Heidelberg, Heidelberg, Germany; 3 Ulm University and Ulm University Medical Center, Ulm, Germany; 4 University of Nairobi, Nairobi, Kenya; 5 Department of Medical Biotechnology & Research Center of Advanced Technologies in Medicine, Torbat Heydariyeh University of Medical Sciences, Torbat Heydariyeh, Iran; 6 Moi Eldoret University, Eldoret, Kenya; 7 Department of Experimental, Diagnostic, and Specialty Medicine Bologna University Medical School, S. Orsola Malpighi Hospital, Bologna and Euro-Mediterranean Institute of Science and Technology (IEMEST), Palermo, Italy; 8 Jomo Kenyatta University of Agriculture and Technology, Nairobi, Kenya; 9 Pathodiagnostik Lab, Berlin, Germany

**Keywords:** Lymphoma, Haematological cancer

## Abstract

*MYC* is the most altered oncogene in human cancer, and belongs to a large family of genes, including *MYCN* and *MYCL*. Recently, while assessing the degree of correlation between *MYC* gene rearrangement and MYC protein expression in aggressive B-cell lymphomas, we observed few Burkitt lymphoma (BL) cases lacking MYC protein expression despite the translocation involving the *MYC* gene. Therefore, in the present study we aimed to better characterize such cases. Our results identified two sub-groups of MYC protein negative BL: one lacking detectable MYC protein expression but presenting MYCN mRNA and protein expression; the second characterized by the lack of both MYC and MYCN proteins but showing *MYC* mRNA. Interestingly, the two sub-groups presented a different pattern of SNVs affecting *MYC* gene family members that may induce the switch from *MYC* to *MYCN*. Particulary, MYCN-expressing cases show MYCN SNVs at interaction interface that stabilize the protein associated with loss-of-function of MYC. This finding highlights MYCN as a reliable diagnostic marker in such cases. Nevertheless, due to the overlapping clinic, morphology and immunohistochemistry (apart for MYC versus MYCN protein expression) of both sub-groups, the described cases represent bona fide BL according to the current criteria of the World Health Organization.

## Introduction

*MYC*, a proto-oncogene located on chromosome 8q24, is the most commonly altered oncogene in human cancer^1,2^. The encoded protein (MYC) is a multifunctional, nuclear phosphoprotein that plays a key role in cell cycle progression, apoptosis, cellular differentiation, and metabolism^3^. It functions as a transcription factor that regulates expression of about 15% of all human genes^3^ through binding on enhancer box sequences (E-boxes) and recruiting histone acetyl-transferases (HATs). In addition to its role as a classical transcription factor, *MYC* also acts to regulate global chromatin structure by modifying histone acetylation both in gene-rich regions and at sites far from known genes^4^. A strict check of MYC expression is physiologically accomplished by controlling it at multiple levels, i.e. transcription, translation, and mRNA and protein stability. *MYC* belongs to a large family of genes, also including *MYCN* and *MYCL1* in human^5^. Despite the *MYC* family members display notable differences in the patterns of expression, they function in a similar manner and have similar genomic structures. In particular, the the *MYC* and *MYCN* loci are similarly organized and both genes comprise three exons. Most of the first exon and the 3′ portion of the third exon contain untranslated regions that carry transcriptional or post-transcriptional regulatory sequences^5,6^. Since, they present high homology in their sequences and protein binding sites and largely share their target genes, they can compensate and substitute for each other in both physiological and pathological conditions^5–8^. Previous studies demonstrated a cross regulating expression of MYC family members; in particular, it has been shown that MYC and MYCN reciprocally control their expression via regulatory loops and via repressing each other at defined promoter sites^9–12^.

Concerning human lymphoid neoplasms, MYC is typically expressed in Burkitt lymphoma (BL), as a consequence of the t(8;14)(q24;q32) translocation or its variants. Moreover, a variable proportion of plasmablastic lymphomas (PBLs), diffuse large B-cell lymphomas (DLBCLs), mantle cell lymphomas (MCLs), and plasma cell myelomas express MYC^13^. In contrast, MYCN expression has not been systematically studied so far in lymphoid neoplasms. It has been recently shown that MYC and MYCN are both required for hematopoietic stem cell (HSC) proliferation, metabolic growth, differentiation, long-term self-renewal activity, and survival^14^. Moreover, MYCN is expressed in self-renewing, quiescent stem cells, also including the hematopoietic ones that switch to higher MYC expression in transit-amplifying progenitors that further differentiate^14–16^.

Interestingly, in a previous study on the standardization of MYC protein expression by immunohistochemistry (IHC) and its correlation with *MYC* gene rearrangements by fluorescent in situ hybridization (FISH) in BL and DLBCL, we detected few BL cases lacking MYC protein expression despite carrying a translocation involving the *MYC* gene^17^.

Therefore, in the present study we aimed to (1) better characterize such BL cases lacking MYC protein expression, (2) evaluate whether a cross-talk between the *MYC* gene family members does also exist in BL, and (3) explore the genetic landscape of this subset of BL cases.

## Materials and methods

### Cases selection, immunophenotype and FISH

We studied 92 morphologically and immunophenotypically typical BL cases (82 pediatric and 10 adult; median age: 12 years (range 3–79)). All cases have been diagnosed according to the updated World Health Organization (WHO) classification of tumors of haematopietic and lymphoid tissues^12^. The cases were retrieved from the archives of four institutions, namely Siena University Hospital (Italy, *n* = 8), Pathodiagnostik Laboratory Berlin (Germany, *n* = 4), Nairobi University (Kenya, *n* = 50), and Moi University, Eldoret (Kenya, *n* = 30) and considering their regional derivation by definition included 12 sporadic and 80 endemic samples. Before enrolling the cases in this study, they were re-evaluated by expert hematopathologists (LL, HS) and diagnoses were confirmed by morphology on histological slides stained with haematoxylin and eosin (H&E) or giemsa, and by immunophenotyping. The main clinical features of our cohort are summarized in Supplementary Table 1. All the procedures were carried out automatically on representative paraffin sections from each case by Bench Mark Ultra (Ventana, Monza, Italy) using extended antigen retrieval and with DAB as chromogen. MYC detection was performed by exploiting the clone Y69 (Ventana and Epitomics, Germany)^18^. For MYCN we employed the ab198912 (Abcam, Cambridge, UK). Both antibodies produced a strictly nuclear staining. As positive control, a BL case expressing MYC protein at high level and characterized by *MYC* gene rearrangement by FISH analysis, was used; for MYCN, human brain tissue was used as control. Negative control was provided by replacing the two antibodies with non-immune mouse serum. The intensity of staining and the percentage of positive neoplastic cells for MYC and MYCN were evaluated by two hematopathologists (MRA and SL) independently and scored according to previous published data^17^. Scoring was evaluated on strongly stained nuclei and in hot spot areas, if present^19^. FISH analysis for *MYC* gene rearrangements was performed using break-apart probes in all cases (ZytoLight SPEC MYC Dual Color Break Apart Probe, Bio-Optica, Germany) following the manufacturer’s instructions. In addition, we used dual-fusion probes (ZytoLight SPEC MYC/IGH Dual Color Dual Fusion Probe, Bio-Optica, Germany) in *MYC* translocation negative samples and in the MYC protein-negative cases following the manufacturer’s instructions. The IGH-MYC negative cases were further evaluated by IGK-MYC and IGL-MYC probes (Supplementary Table 1)^20^. FISH analysis of chromosome 2p24/CEP2 for *MYCN* amplification [Vysis LSI MYCN (2p24) Spectrum Green/Vysis CEP2 Spectrum Orange Probe, Abbott, USA] was also performed as previously described^21^. A *MYCN* break apart FISH assay was applied, containing four clones, which flank the *MYCN* gene, RP11-105P20 (spectrum green), RP11-422A6 (spectrum green), RP11-355H10 (spectrum orange) and RP11-744F11 (spectrum orange). For each specimen, at least 100 intact non-overlapping non nuclei were analyzed manually on a Leika DM 600B (Leica Microsystems, Switzerland) or Zeiss fluorescence microscope equipped with DAPI, SpectrumGreen, SpectrumOrange filters. DNA preparation from bacterial clones and fluorescent labeling were performed following recently described protocols (Supplementary Table 1)^22^. Appropriate negative and positive controls were used^22^. In situ hybridization (ISH) for Epstein-Barr virus encoded RNAs (EBER) was carried out in each sample on 5 mm thick section as previously described^23–25^. A control slide prepared from a paraffin-embedded tissue block containing metastatic nasopharyngeal carcinoma in a lymph node accompanied each hybridization run.

The study was approved by the institutional ethical committees of the institutions submitting the cases, and written permission and informed consent have been obtained before samples collection in accordance with the Declaration of Helsinki.

### RNA extraction

RNA was extracted from FFPE sections of primary tumors and reactive lymph nodes using the FFPE RNA Easy kit (Qiagen, CA), and from cell lines using the RNA Easy Kit (Qiagen, CA), according to the manufacturer’s instructions. The amount and quality of RNA were evaluated by measuring the OD at 260 nm and the 260/230 and 260/280 ratios using a Nanodrop spectrophotometer (Celbio, Milan, Italy). The quality of RNA was also checked using a Bioanalyzer 2100 (Agilent, CA, USA).

### In situ detection of MYC and MYCN mRNA by RNAscope assay

RNA ISH was performed to investigate the expression of *MYC* gene family members at mRNA level. RNAscope 2.5 HD Red Detection Kit (Advanced Cell Diagnostics, Hayward, CA, USA) and RNAscope Probes for MYC and MYCN mRNA (Hs-MYC, Cat # 311761; Hs-MYCN, Cat # 417501) were applied, according to the manufacturer’s instructions^26^. Briefly, sections of formalyn-fixed paraffin-embedded (FFPE) tissue were baked for 1 h at 60 °C prior to use. After de-paraffinization and dehydration, the tissues were air dried and treated with a peroxidase blocker before boiling in a pre-treatment solution for 10 min. Protease was then applied for 30 min at 40 °C. Target probes were hybridized for 2 h at 40 °C, followed by a series of signal amplification and washing steps. Probes are hybridized and followed by a cascade of signal amplification which enhances the signal for low expressing gene and mRNA present in archived samples and partial degraded specimens. Hybridization signals were detected by chromogenic reactions using Fast Red. mRNA staining signal was identified as cytoplasm and nuclear red punctate dots. Each sample was quality controlled for mRNA integrity with a probe specific to the housekeeping *PPIB* mRNA. Negative control background staining was evaluated by using a probe specific to bacterial *dapB* gene; all cases did not show any signals in the neoplastic tissue, therefore they were included in the analysis.

### Reverse transcription-quantitative PCR (RT-qPCR)

In primary tumors, the expression of *MYC* has been investigated by RT-qPCR using two different approaches: by using primers designed with the help of Primer-BLAST service (Supplementary Table 2) and by using specific Taqman probes for *MYC* gene detecting all the three *MYC* gene exons (Cat. # 4331182, ThermoFisher Scientific, USA). This approach aimed to rule out possible technical failures due to splicing variants in these cases. The measures obtained from all assays were used to calculate the mean value of MYC mRNA. Thus, the resulting *MYC* expression is a merge of all exons studied. MYCN mRNA has been checked by using the specific Taqman probe (Cat. # 4331182, ThermoFisher Scientific, USA) according to the manufacturer’s instructions. Four endogenous controls (hypoxanthine-guanine phosphoribosyltransferase, *HPRT*; Phosphoglycerate kinase, *PGK*; Beta-2-Microglobulin, *b2m*; TATA-Box Binding Protein, *TBP*) were included in the experiment. Considering that *HPRT* housekeeping gene showed the higher and more constant expression in all our cases, we selected it for relative quantification of each target gene.

Reactive lymph nodes have been used as control and the relative expression is expressed as 2^−ΔΔCt^.

### Next generation sequencing (NGS)

Targeted NGS of 409 cancer related genes was performed on 40 ng tumoral DNA using the IonAmpliSeq Comprehensive Cancer Panel (Thermo Fisher Scientific, USA) according to the manufacturer’s protocol. Alignment, variant calling and filtering were performed with Ion Reporter 4.4 (Thermo Fisher Scientific, USA). The following filter chain was used: “Location in utr_3, splicesite_3, exonic, splicesite_5, utr_5” in, “variant effect in stoploss, nonsense, missense, frameshift Insertion, non-frameshift Insertion, non-frameshift Block Substitution, frameshift Deletion, non-frameshift Deletion, frameshift Block Substitution. The base coverage was minimum 60.80% and maximum 70.36%, with average equal to 65.71%. The mean of reads with >Q20 was equal to 92.59%.

### Sanger sequencing

To further explore the MYCN locus, primers were designed to amplify 200 bp fragments covering all exons of *MYCN* gene. PCR products were purified and subjected to Sanger sequencing in two reactions, one with the forward and one with the reverse primer (Thermo Fisher Scientific, USA, Catalog # A15629, A15630).

### Functional in vitro studies

The human BL cell line Namalwa (ATCC CRL-1432) and a human B lymphoblastoid cell line (LCL; GK-5 (ATCC® CRL-183)) were used to perform the in vitro experiments. Namalwa was characterized by *MYC*-rearrangements and strong expression of MYC mRNA. The LCL has been investigated to better appreciate the effect of *MYC* silencing. Both cell lines were EBV-positive. Briefly, cells were cultured in RPMI-1640 medium supplemented with 10% Fetal Bovine Serum (FBS), 1% l-glutamine, 1% penicillin/streptomycin (CARLO ERBA Reagents, Milan, Italy), with 5% CO_2_, at 37 °C. Transient transfections were performed by nucleofection, using an Amaxa Nucleofector device (Lonza, Cologne, Germany), program A23 and solution V (Lonza, Cologne, Germany) as a buffer solution, following the manufacturer’s instructions. 5 × 106 cells were transfected with 0.5 and 1 μg small interfering RNA (siRNA) targeting MYC, esiRNA human MYC (MISSION esiRNA Human MYC (esiRNA1), Sigma Aldrich, St. Louis, USA) or with 1 μg esiRNA targeting RLUC (esiRNA1) used as negative control of gene inhibitor (MISSION esiRNA RLUC (esiRNA1), Sigma Aldrich, St. Louis, USA); transfection solution was used as a mock. Transfection efficiency was assessed transfecting 2 μg of pmaxGFP and detecting both fluorescence and cell viability by flow cytometry; RNA was extracted 48 and 72 h after nucleofection. *MYC* and *MYCN* expression was checked by RT-qPCR as described above.

### Statistical analyses

Statistical analyses were performed using IBM SPSS Statistics 20.0 (IBM, Armonk, NY, USA) and Prism (GraphPad Softwares, La Jolla, CA, USA). ANOVA, unpaired T-tests, and linear regression were used for continuous variable analysis. Chi-square was used for non-continuous variable analysis. Two-sided tests were used in all calculations. The limit of significance for all analyses was defined as *p* < 0.05.

### Modeling predicted effects

We annotated SNVs on *MYCN* and *MYC* canonical amino acid sequence through Ensembl Variant Effect Predictor (VEP)^27^ and graphically displayed through the lollipos software^28^. We used ELM (http://elm.eu.org/)^29^ to predict mutation effects on linear motives, while we employed Mechismo (http://mechismo.russelllab.org/)^30^, using default settings^31^ to predict mutations effects at 3D interaction interfaces. Similarly to what we did in a previous study^32^, we analyzed SNVs in the exon 2 of *MYC* gene for enrichment in phosphosite area, considered a window of −/−4 aminoacids close to phosphosite residues. Frequency of observed SNVs in phosphosite was obtained dividing the observed number of SNVs in phosphosite area (12 and 9 for MYCN positive and MYCN negative, respectively) for its length (*n* = 78), while the expected frequency was done by dividing the total number of SNVs (34 and 31, respectively) in exon 2 for its length (*n* = 252) (Supplementary Tables 3, 4). For the statistical analysis Fisher’s exact test was done.

## Results

### Rare BL cases lack MYC expression despite MYC gene translocation

**Fig. 1:**
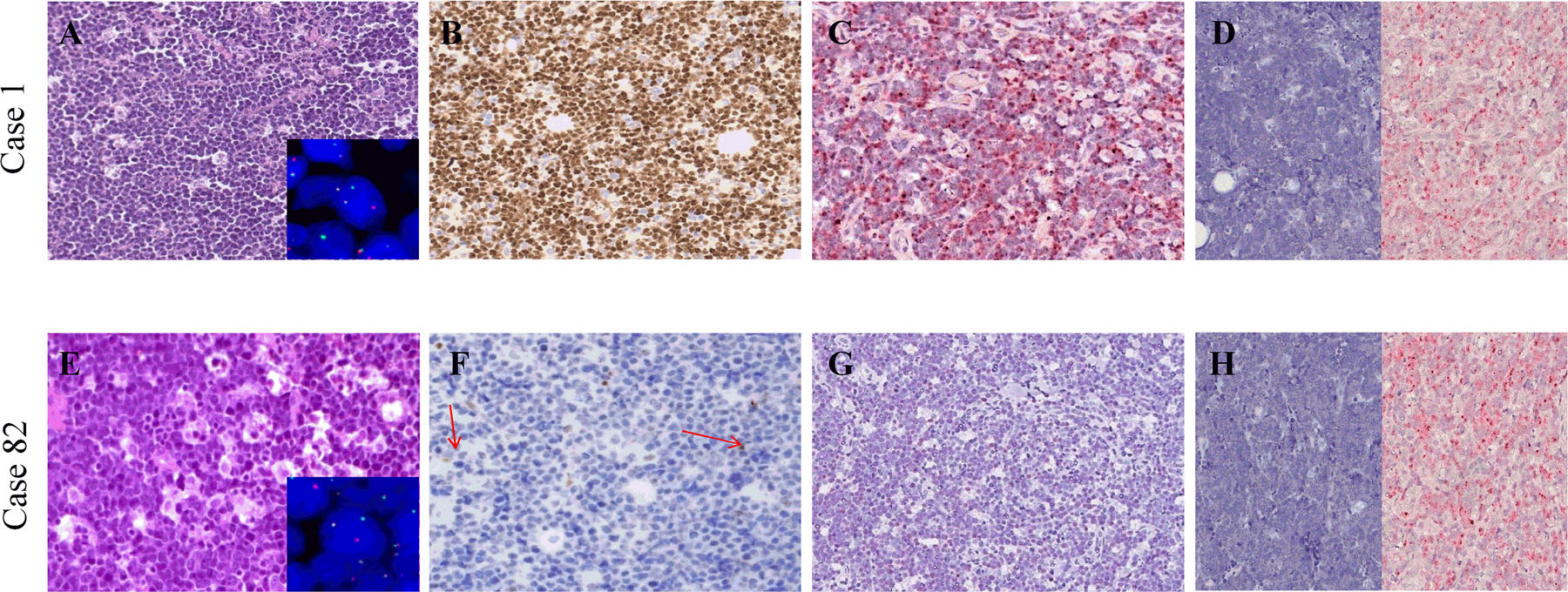
Morphology, immunophenotype and cytogenetics of our cohort. **a** A BL case with the typical morphology carrying MYC gene translocation (inset) and expressing MYC at protein and mRNA level. **b** An example of those cases that despite MYC gene translocation (inset), did not express MYC at protein level and showed a heterogeneous staining for MYC mRNA; these cases presented the characteristic cohesive growth, squared-off cytoplasm and starry-sky appearance; scattered positive non-neoplastic cells (red arrows) served as internal control to ensure a successful immunohistochemical reaction. A–B, from left to right: haematoxyin and eosin (H&E), MYC protein staining (brown chromogen; Y69 clone), RNAscope assay for MYC mRNA (red chromogen). dapB and PPIB probes were applied as negative and positive controls, respectively. A–B, Original magnification (O.M.): ×20.

Ninety out of ninety-two cases (98%) showed a translocation involving *MYC* gene detectable by FISH analysis (Supplementary Table 1). At immunohistochemistry, eighty-three out of ninety-two cases (90%) did present with intense and diffuse nuclear MYC protein expression in more than 80% of neoplastic cells (Fig. 1a), including the two cases lacking an identifiable *MYC* gene translocation by commercial probes, suggesting the presence of a *MYC* juxtaposition to one of the not tested light chain loci or an alternative means of MYC activation, like cryptic insertion of *MYC* into IG loci^20,33–36^.

**Fig. 2:**
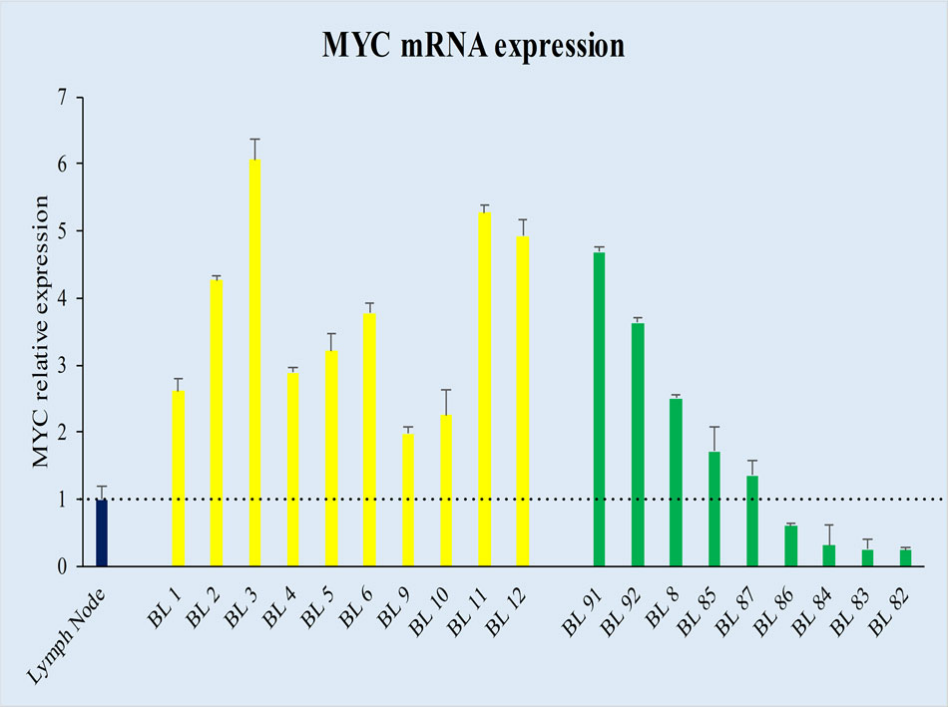
Comparison of MYC protein positive with MYC protein-negative BL cases in terms of mRNA by RT-qPCR. The merge of measures obtained by applying different assays detecting all MYC exons demonstrated a heterogeneous MYC mRNA expression (*y* axis) among the different samples (*x* axis). The threshold is represented by black dotted line. Specifically, some BL cases without MYC protein expression showed a MYC mRNA expression overlapping that detected in MYC protein-positive cases; whereas, others showed a very low expression with a value below that detected in the normal lymph node used as control (lymph node in blue, MYC protein-positive cases in yellow, MYC protein-negative cases in green).

Remarkably, 9/90 (7 endemic BL-eBL, 2 sporadic BL-sBL) cases carrying a translocation involving *MYC* gene did not express MYC at the protein level, showing a weak positivity in only 0–5% cells (Fig. 1b; Table 1). All the nine cases presented a *MYC* gene translocation by break apart probes, seven out of nine showed an MYC/IGH fusion; the 2 cases in which the *IGH/MYC* fusion probes did not demonstrated a juxtaposition of *MYC* to *IGH*, were tested by IGK and IGL probes, revealing a translocation involving the light chain lambda gene locus (Table 1). To evaluate whether the lack of MYC protein observed in 9/90 cases with *MYC* breakpoint was related to transcriptional or post-transcriptional issues, MYC mRNA was investigated by RNAscope and RT-qPCR. Strong expression of MYC mRNA visualized as punctate red dot signals was detected in BL cases characterized by a marked expression of MYC protein by immunohistochemistry (Fig. 1a), whereas the cases with the absence of MYC protein showed a heterogeneous staining for MYC mRNA with few cases being almost completely negative (Fig. 1b). These findings overlapped the RT-qPCR results (Fig. 2) that showed a heterogeneous MYC mRNA level ranging from 1.98 to 6.06 for MYC protein-positive BL cases and from 0.24 to 4.68 for MYC protein-negative specimens (Fig. 2).

**Table 1:** Clinical data and representative mutation sites located on MYC and MYCN genes.

Case	Age	Sex	Epidemiology	Biopsy site	EBER	*MYC* FISH			PROTEIN		SNVs position	AA switch
						MYC B.A.	MYC-IGH	MYC-IGK/IGL	MYC	MYCN		
**BL 8**	14	F	endemic	lymph node	−	+	+	n.p.	−	+	g.chr2:16086085G>A	V421I
**BL 82**	7	M	endemic	ileum	−	+	−	+ (IGL)/-(IGK)	−	+	g.chr2:16086115C>T g.chr8:128753056C>T	H431Y P406L
**BL 83**	8	M	endemic	soft tissue	+	+	+	n.p.	−	−	g.chr8:128750542T>G g.chr8:128750610G>T g.chr8:128750607G>A	Y27D Q49H Q48Q
**Bl 84**	9	F	endemic	maxilla	+	+	+	n.p.	−	+	g.chr2:16086046G>A g.chr8:128752981G>A	V408M R381K
**BL 85**	11	M	endemic	oral cavity	+	+	+	n.p.	−	+	g.chr2:16086104C>T	T427I
**BL 86**	4	F	endemic	lymph node	+	+	+	n.p.	−	+	g.chr2:16086208C>T g.chr8:128753079G>A	R462W A414T
**BL 87**	10	M	endemic	maxilla	+	+	−	+ (IGL) / -(IGK)	−	+	g.chr2:16086076G>A g.chr8:128753064G>A	A418T V409I
**BL 91**	45	M	sporadic	stomach	−	+	+	n.p.	−	−	g.chr8:128750540A>G g.chr8:128750543A>C	N26S Y27S
**BL 92**	79	F	sporadic	vagina	−	+	+	n.p.	−	−	g.chr8:128750543A>G	Y27C

### Small subset of BL cases present MYCN expression

**Fig. 3:**
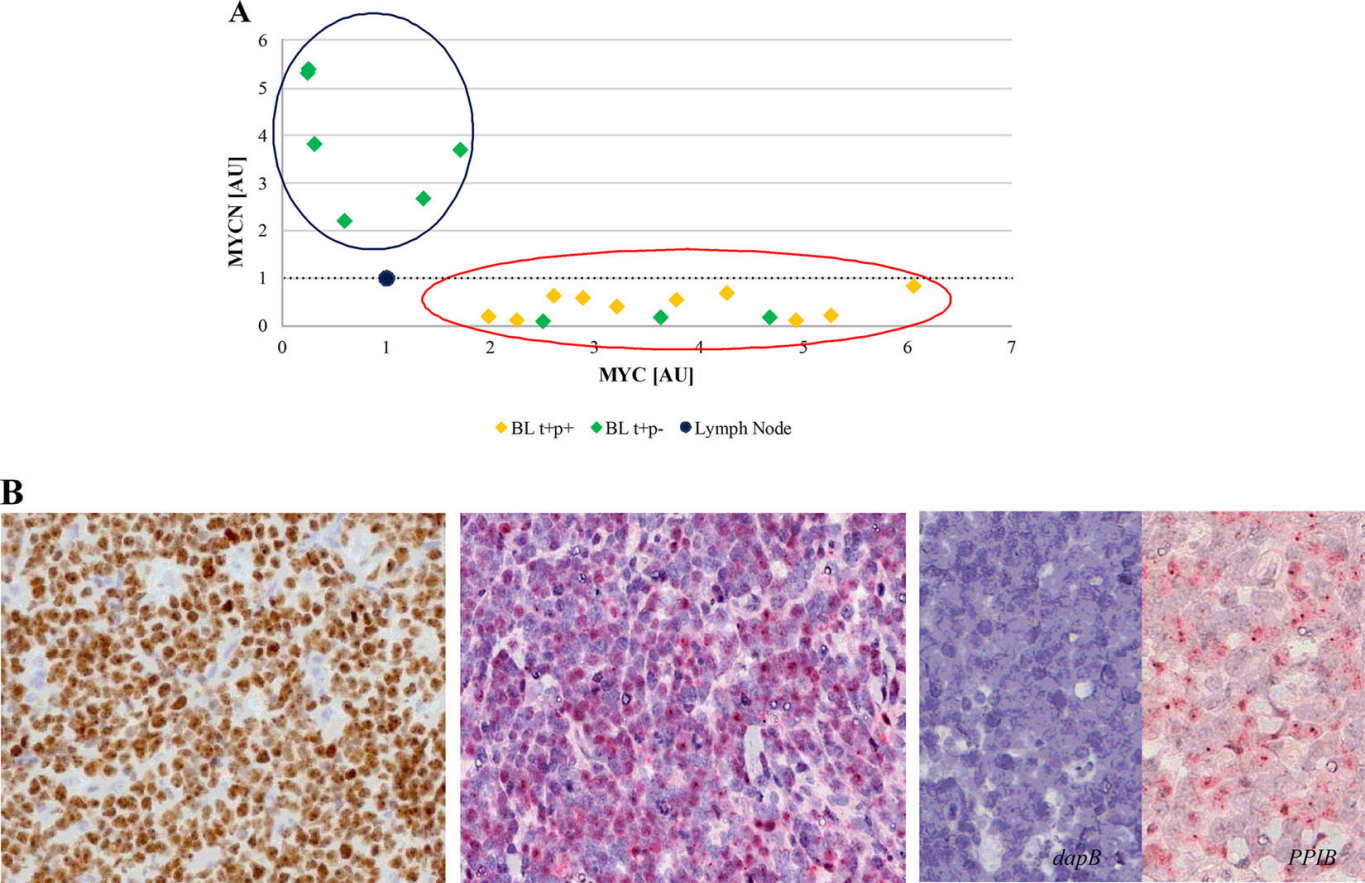
Correlation between MYC and MYCN expression at mRNA by RT-qPCR and protein level by IHC. An almost mutual exclusivity between MYC and MYCN was identified at mRNA and protein level. Specifically, MYCN transcript was clearly detectable almost exclusively in MYC gene/protein-negative cases. **a** The regression analysis comparing the expression of MYC (*x* axis) and MYCN (*y* axis) mRNA identified two different clusters: one consisting of six cases characterized by very low MYC mRNA level and higher MYCN mRNA expression (circle blue); the second contains all the other cases, including the MYC translocation-positive/protein-positive samples and the cases being MYC translocation-positive/protein negative but expressing MYC at mRNA level (circle red). The threshold is represented by black dotted line. **b** Immunohistochemical evaluation and RNAscope assay for MYCN protein and mRNA expression showed a strong MYCN nuclear staining identified respectively as brown and red nuclear signals, in almost 90% of neoplastic cells only in the six MYCN mRNA positive samples. Probes detecting dapB and PPIB were used as negative and positive controls, respectively.

The heterogeneous expression of MYC mRNA and protein level in our cohort raised the question of how cases lacking MYC mRNA and/or protein could maintain a complete BL phenotype. Previous studies have demonstrated a regulatory loop among *MYC* gene family members, specifically, between *MYC* and *MYCN*^9–12,37^. Accordingly, we evaluated MYCN mRNA and protein expression in such BL cases. Interestingly, we detected an almost mutual exclusivity between the expression of the two genes in our series at mRNA level by RNAscope and RT-qPCR, with cases expressing MYCN mRNA lacking MYC mRNA and vice versa (*p* = 0.0003, Student’s *t* test, unpaired). The regression analysis identified two different clusters: one consisting of six cases characterized by very low MYC mRNA level and higher MYCN mRNA expression; the second cluster contains all the other cases, including the typical BLs and those cases characterized by MYC protein negativity but showing MYC mRNA (Fig. 3a). Then we validated our results at protein level by evaluating MYCN protein expression. A strong MYCN nuclear staining in almost 90% of neoplastic cells was detected in the six MYCN mRNA positive samples (Fig. 3b) while the remaining cases showing only MYC mRNA did not express MYCN at protein level. In such cases we investigated all the mechanisms responsible of MYCN over-expression (i.e. amplification, translocation and proviral insertion)^38^. Specifically, by FISH analysis of chromosome 2p24/CEP2, no amplification or translocation of the *MYCN* gene was detected. In addition, sequencing of *MYCN* gene did not provide evidence for a possible proviral insertion (by EBV, cytomegalovirus, HHV8) that could explain an enhanced *MYCN* transcription in the absence of increased copy number. We also investigated the association with EBV by applying EBER-ISH assay. Seventy-three cases (70 eBL, 3 sBL) resulted EBV-positive while nineteen (10 eBL, 9 sBL) were negative. The statistical analysis did not show a significant difference between the MYCN negative (69% EBV-positive) and MYCN positive (66% EBV-positive) cases.

### Genomic analysis supports the presence of two subsets of BL depending on MYCN expression

We then explored the genetic landscape of eight out of the nine MYC protein-negative cases for which enough DNA was available by an ultra-deep sequencing analysis targeted on 409 cancer associated genes. Since we found SNVs affecting genes previously reported in BL and other lymphomas^39–46^ we decide to focus our attention on the mutational landscape of *MYC* family genes. Remarkably, in cases lacking *MYCN* expression, we identified SNVs only in *MYC* gene; specifically, we detected SNVs within the region coding for the N-terminus domain (NTD) of MYC. In particular, we reported SNVs in the MYC gene resulting in amino acid changes at position 27 (chr8:128,750,543A>C, p.Y27S; chr8:128,750,543A>G, p.Y27C; chr8:128,750,542T>G, p.Y27D) of the MYC protein (Supplemenatry Table S3). It is likely that these SNVs, adjacent to N11 polymorphism and within the Y69 target epitope, may interfere with antibody binding and explain the MYC protein-negative staining.

**Fig. 4:**
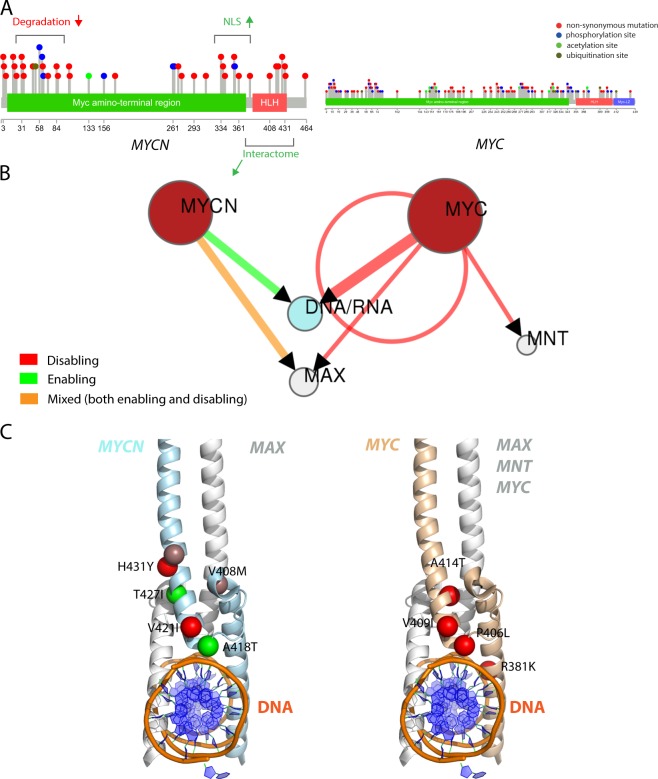
MYC and MYCN non-synonymous mutations comparison. Mutations (red lollipops) annotated on protein primary sequence with additional information regarding post-translational modifications (PTMs) and domain composition. Linear motif annotations have been obtained from ELM (http://elm.eu.org/cgimodel.py?fun=smartResult&userId=QiKSrcdQR9&EXPECT_CUTOFF=100&r=1&bg=on). MYC mutations have been mapped to the canonical (isoform 1 from Swissprot, ID: P01106) protein isoform. Only mutations for MYCN positive cases are displayed. **b** Network representation of predicted effects at 3D interaction interfaces through Mechismo (http://mechismo.russelllab.org/). Predicted disabling, enabling and mixed effects are in indicated by red, green and orange arrows. **c** 3D cartoon representation (PDB ID: 1NKP) of mutations perturbing interaction interfaces. Sphere coloring is the same as for arrows in B. For MYC, we show mutation numberings referred to both the canonical amino acid sequence as well as to the isoform 2.

**Fig. 5:**
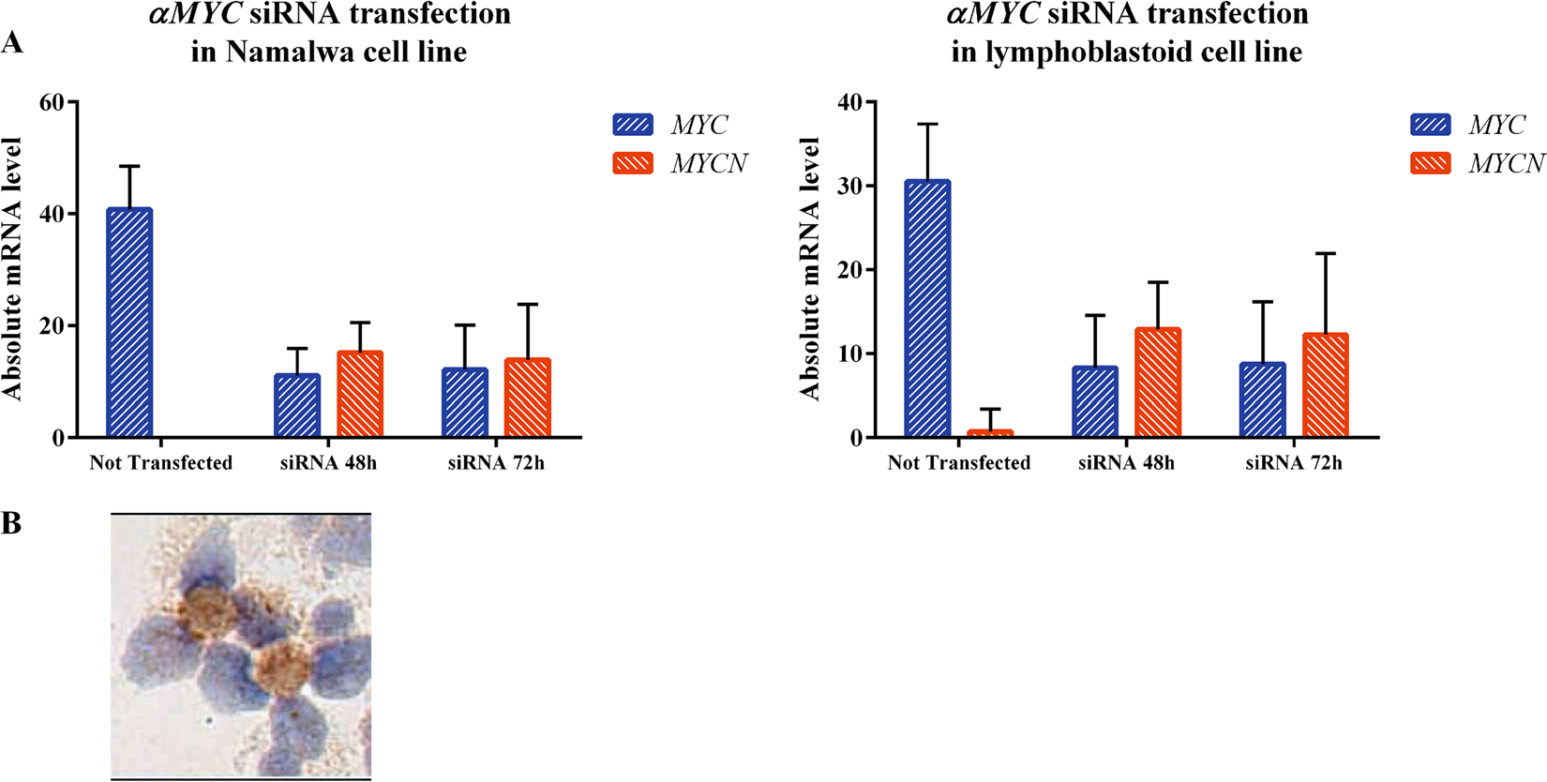
Regulatory loop between MYC and MYCN exists also in Namalwa and lymphoblastoid cell lines. **a** Silencing of MYC mRNA expression by siRNA nucleofection in Namalwa and lymphoblastoid (B) cell lines resulted in a lower expression of MYC mRNA along with higher expression of MYCN. MYC mRNA has been investigated by RT-qPCR applying designed and Taqman primers. **b** Transfected cells collected after MYC silencing showed MYCN protein expression by immunohistochemistry.

On the other hand, MYCN positive samples, carried MYCN SNVs concentrated at the N-and C-terminus of the MYC amino-terminal region and at the helix-loop-helix (HLH) domain (Fig. 4a), where they likely perturb regulatory or functional motives of the protein. Indeed, while the first region (a.a. 20–90) is a segment important for post-translational modifications (PTMs) and binding events (e.g. GSK3, p38 MAPK, WW binding domains and FBXW7) leading to MYCN degradation^47^, the second one (approximately a.a. 300–370), contains nuclear localization sequences (NLSs) (Fig. 4a; http://elm.eu.org/). The overall electrostatic charge switch caused by non-synonymous mutations in MYCN, with nearly 40% of SNVs leading to an increase of positive charge (Supplementary Table 3), is likely to affect the binding and signaling properties mediated by these motives. Along this line, SNVs affecting the C-terminal HLH domain are predicted to positively affect MYCN interactome in these cases, in contrast to MYC, which is overall disabled (Fig. 4b, c). Taken together, MYCN mutations in MYCN expressing cases are predicted to lead to an overall stabilization of its activity, by ultimately perturbing degradation signals while enhancing nuclear localization and mediated interactions. Interestingly, we found no significant enrichment of SNVs in *MYC*’s exon 2 phosphosite regions (Supplemetary Tables 3, 4), which is a hallmark of MYC up-regulation in sporadic BL^32^. Finally, to further support the hypothesis of a cross-talk between MYC and MYCN in BL, we silenced *MYC* gene in BL Namalwa and LCLs cell lines by shRNA and evaluated MYCN mRNA and protein expression after 48 and 72 h. We found that silencing of *MYC* gene results in higher expression of both MYCN mRNA and protein (Fig. 5a). Particularly, after siRNA nucleofection, MYC mRNA levels in Namalwa cell line dropped down from 40.79 arbitrary units (AU) to 11.03 AU at 48 h and 12.11 AU at 72 h, while *MYCN* increased from 0 to 15.2 at 48 and 13.89 at 72 h (*p* < 0.0001). In LCLs cell line *MYC* dropped down from 30.5 to 8.28 at 48 h and 8.75 at 72, while *MYCN* raised from 0.75 to 12.9 and 12.26 (48 and 72 h respectively). Consistently, MYCN protein expression was recorded after transfection by RT-qPCR (Fig. 5b).

## Discussion

In this paper we describe a rare subset of BL cases characterized by the lack of MYC protein expression and the presence of MYCN protein. These rare cases, despite FISH analysis documenting *MYC* gene translocation to one of the immunoglobulin loci, lacked MYC protein expression and expressed another MYC family member, MYCN. Noteworthy, we observed an inverse correlation between the expression of MYC and MYCN at mRNA and protein level. It has been previously demonstrated that MYCN is able to compensate MYC activity in neuroblastoma cell lines and primary tumors, a mutual regulatory loop existing between them^7–13^. NGS analysis showed a different mutational fingerprint of *MYC* family genes in cases expressing or not MYCN at mRNA and protein level. Specifically, *MYCN* negative cases presented SNVs in *MYC* genes localized within the region coding for the NTD. It is conceivable that such SNVs prevented an effective antigen-antibody reaction, as recently reported^34^, thus determining the negative results at immunohistochemistry. By contrast, in MYCN positive samples analysis of MYCN SNVs at linear motives and interaction interfaces suggests converging effects towards an overall stabilization of protein activity, by either perturbation of degradation signals, enhancement of nuclear localization, or interactome stabilization. On the other hand, multiple somatic mutations affecting *MYC* suggest an overall loss-of-function phenotype, differently from sporadic BL, where SNVs cluster at exon 2 phosphosite regions leading to *MYC* up-regulation^32^. It is intriguing to speculate that the SNVs present in *MYC* and *MYCN* in the respective cases may activate and switch on *MYCN* gene and simultaneously switch off *MYC* gene by inducing their regulatory loop^48^. Interestingly, we found that silencing of *MYC* gene results in higher expression of both MYCN mRNA and protein. In particular, after siRNA nucleofection, MYC mRNA levels decreased while MYCN ones increased and MYCN protein expression was detectable in cell lines.

In conclusion, it is conceivable that *MYCN* determined molecular effects, in terms of transcriptional regulation, similar to those exerted by *MYC* in BL cells thanks to a cross-talk between the two genes involving a significant number of targets shared by MYC and MYCN^9–11,15,16^. This mirrors what is already known in neuroblastoma cell lines and primary tumors in which the expression of MYC and MYCN are mutually exclusive^9,11,15,16^. Remarkably, we have yet only detected the switch from MYC to MYCN expression in MYC-translocation-positive BL in the pediatric age group and only in eBL. Nevertheless, the genetic composition of the tumors suggests that the switch occurs in the presence and probably subsequently to the *IG-MYC* translocation. Therefore and considering that the clinical presentation, morphologic appearance and immunohistochemical profile of these MYC protein negative/MYCN protein-positive *MYC*-translocated tumors is not different from MYC protein-positive BL we think that this does not affect the diagnostic work-up as such cases might be easily diagnosed as BL based on current WHO criteria^13^.

## Supplementary information

Supplementary Table 1

Supplementary Table 2

Supplementary Table 3

Supplementary Table 4
