# Supplementary material for: Molecular switch from MYC to MYCN expression in MYC protein negative Burkitt lymphoma cases
Source: Blood Cancer J. 2019 Nov 20;9(12):91. doi: 10.1038/s41408-019-0252-2 (PMC6868231; doi:10.1038/s41408-019-0252-2)
Supplement: Supplementary file 1 — Supplementary Table 1 [file 41408_2019_252_MOESM1_ESM.docx]

| **BL** | **Age** | **Sex** | **BL subtype** | **Site of biopsy** | **EBER** | **HIV status** | ***MYC***  **FISH**  **B.A.** | ***MYC-***  ***IGH***  **FISH** | ***MYC-***  ***IGK/IGL***  **FISH** | **MYC**  **PROTEIN** | **MYCN**  **PROTEIN** |
| --- | --- | --- | --- | --- | --- | --- | --- | --- | --- | --- | --- |
| **1** | 40 | F | sBL | Inguinal lymph node | - | - | + | n.p. | n.p. | + | - |
| **2** | 18 | M | sBL | Ileum | - | - | + | n.p. | n.p. | + | - |
| **3** | 3 | M | eBL | Stomach | - | n.a. | + | n.p. | n.p. | + | - |
| **4** | 5 | M | eBL | Ileum | - | n.a. | + | n.p. | n.p. | + | - |
| **5** | 38 | F | sBL | Lymph node | - | - | + | n.p. | n.p. | + | - |
| **6** | 40 | F | sBL | Bone marrow | - | - | + | n.p. | n.p. | + | - |
| **7** | 13 | M | eBL | Ileum | - | - | + | n.p. | n.p. | + | - |
| **8** | 14 | F | eBL | Lymph node | - | n.a. | + | + | n.p. | - | + |
| **9** | 20 | M | sBL | Lymph node | - | - | - | - | n.p. | + | - |
| **10** | 6 | M | eBL | Maxilla | + | n.a. | - | - | n.p. | + | - |
| **11** | 7 | F | eBL | Oral cavity | - | n.a. | + | n.p. | n.p. | + | - |
| **12** | 14 | M | sBL | Lymph node | + | - | + | n.p. | n.p. | + | - |
| **13** | 12 | M | eBL | Oral cavity | - | n.a. | + | n.p. | n.p. | + | - |
| **14** | 11 | M | eBL | Lymph node | + | n.a. | + | n.p. | n.p. | + | - |
| **15** | 9 | M | eBL | Abdomen mass | + | n.a. | + | n.p. | n.p. | + | - |
| **16** | 5 | M | eBL | Lymph node | + | n.a. | + | n.p. | n.p. | + | - |
| **17** | 6 | M | eBL | Lymph node | + | - | + | n.p. | n.p. | + | - |
| **18** | 4 | M | eBL | Mesenteric  lymph node | + | n.a. | + | n.p. | n.p. | + | - |
| **19** | 12 | M | eBL | Abdomen mass | - | n.a. | + | n.p. | n.p. | + | - |
| **20** | 21 | M | eBL | Thyroid | + | - | + | n.p. | n.p. | + | - |
| **21** | 16 | M | sBL | Abdomen mass | - | - | + | n.p. | n.p. | + | - |
| **22** | 12 | M | eBL | Lymph node | + | - | + | n.p. | n.p. | + | - |
| **23** | 10 | F | eBL | Ovary | + | - | + | n.p. | n.p. | + | - |
| **24** | 8 | M | eBL | Ovary | + | - | + | n.p. | n.p. | + | - |
| **25** | 9 | F | eBL | Ileum | + | n.a. | + | n.p. | n.p. | + | - |
| **26** | 8 | M | eBL | Soft tissues | + | n.a. | + | n.p. | n.p. | + | - |
| **27** | 10 | M | eBL | Neck | + | - | + | n.p. | n.p. | + | - |
| **28** | 11 | F | eBL | Abdomen mass | + | n.a. | + | n.p. | n.p. | + | - |
| **29** | 10 | M | eBL | Abdomen mass | + | n.a. | + | n.p. | n.p. | + | - |
| **30** | 8 | F | eBL | Ovary | + | n.a. | + | n.p. | n.p. | + | - |
| **31** | 9 | M | eBL | Ileum | + | n.a. | + | n.p. | n.p. | + | - |
| **32** | 11 | M | eBL | Soft tissue | + | n.a. | + | n.p. | n.p. | + | - |
| **33** | 7 | F | eBL | Ovary | + | n.a. | + | n.p. | n.p. | + | - |
| **34** | 10 | M | eBL | Neck | + | n.a. | + | n.p. | n.p. | + | - |
| **35** | 9 | F | eBL | Pericardium | + | n.a. | + | n.p. | n.p. | + | - |
| **36** | 10 | M | eBL | Spleen | + | n.a. | + | n.p. | n.p. | + | - |
| **37** | 6 | M | eBL | Orbit | + | n.a. | + | n.p. | n.p. | + | - |
| **38** | 4 | M | eBL | Ileum | + | n.a. | + | n.p. | n.p. | + | - |
| **39** | 6 | M | eBL | Abdomen mass | + | n.a. | + | n.p. | n.p. | + | - |
| **40** | 11 | M | eBL | Oral cavity | + | n.a. | + | n.p. | n.p. | + | - |
| **41** | 7 | M | eBL | Kidney | + | - | + | n.p. | n.p. | + | - |
| **42** | 8 | F | eBL | Ovary | + | - | + | n.p. | n.p. | + | - |
| **43** | 11 | M | eBL | Soft tissue | + | n.a. | + | n.p. | n.p. | + | - |
| **44** | 9 | M | eBL | Ileum | + | n.a. | + | n.p. | n.p. | + | - |
| **45** | 7 | M | eBL | Bone marrow | + | + | + | n.p. | n.p. | + | - |
| **46** | 5 | M | eBL | Orbit | + | n.a. | + | n.p. | n.p. | + | - |
| **47** | 7 | M | eBL | Neck | + | + | + | n.p. | n.p. | + | - |
| **48** | 9 | M | eBL | Lymph node | + | n.a. | + | n.p. | n.p. | + | - |
| **49** | 6 | M | eBL | Neck | + | n.a. | + | n.p. | n.p. | + | - |
| **50** | 11 | M | eBL | Testis | + | n.a. | + | n.p. | n.p. | + | - |
| **51** | 13 | M | eBL | Ileum | + | + | + | n.p. | n.p. | + | - |
| **52** | 10 | F | eBL | Ovary | + | n.a. | + | n.p. | n.p. | + | - |
| **53** | 9 | F | eBL | Abdomen mass | + | n.a. | + | n.p. | n.p. | + | - |
| **54** | 12 | M | eBL | Maxilla | + | - | + | n.p. | n.p. | + | - |
| **55** | 10 | F | eBL | Ileum | + | - | + | n.p. | n.p. | + | - |
| **56** | 9 | M | eBL | Neck | + | n.a. | + | n.p. | n.p. | + | - |
| **57** | 14 | F | eBL | Ovary | + | n.a. | + | n.p. | n.p. | + | - |
| **58** | 11 | F | eBL | Neck | + | n.a. | + | n.p. | n.p. | + | - |
| **59** | 7 | F | eBL | Maxilla | + | n.a. | + | n.p. | n.p. | + | - |
| **60** | 10 | F | eBL | Abdomen mass | + | n.a. | + | n.p. | n.p. | + | - |
| **61** | 9 | M | eBL | Neck | + | n.a. | + | n.p. | n.p. | + | - |
| **62** | 12 | F | eBL | Ovary | + | n.a. | + | n.p. | n.p. | + | - |
| **63** | 9 | F | eBL | Maxilla | + | n.a. | + | n.p. | n.p. | + | - |
| **64** | 8 | M | eBL | Ileum | + | n.a. | + | n.p. | n.p. | + | - |
| **65** | 7 | M | eBL | Lymph node | + | n.a. | + | n.p. | n.p. | + | - |
| **66** | 6 | F | eBL | Ovary | + | n.a. | + | n.p. | n.p. | + | - |
| **67** | 8 | F | eBL | Ovary | + | n.a. | + | n.p. | n.p. | + | - |
| **68** | 9 | M | eBL | Ileum | + | n.a. | + | n.p. | n.p. | + | - |
| **69** | 5 | M | eBL | Stomach | + | n.a. | + | n.p. | n.p. | + | - |
| **70** | 7 | F | eBL | Maxilla | + | n.a. | + | n.p. | n.p. | + | - |
| **71** | 8 | F | eBL | Neck | + | n.a. | + | n.p. | n.p. | + | - |
| **72** | 6 | M | eBL | Lymph node | + | n.a. | + | n.p. | n.p. | + | - |
| **73** | 9 | M | eBL | Abdomen | + | n.a. | + | n.p. | n.p. | + | - |
| **74** | 5 | F | eBL | Soft tissues | + | n.a. | + | n.p. | n.p. | + | - |
| **75** | 6 | F | eBL | Maxilla | + | + | + | n.p. | n.p. | + | - |
| **76** | 45 | M | sBL | Neck | + | + | + | n.p. | n.p. | + | - |
| **77** | 54 | F | sBL | Lymph node | + | + | + | n.p. | n.p. | + | - |
| **78** | 10 | F | eBL | Ileum | + | n.a. | + | n.p. | n.p. | + | - |
| **79** | 3 | F | eBL | Lymph node | + | n.a. | + | n.p. | n.p. | + | - |
| **80** | 4 | M | eBL | Maxilla | + | n.a. | + | n.p. | n.p. | + | - |
| **81** | 14 | F | eBL | Breast | + | n.a. | + | n.p. | n.p. | + | - |
| **82** | 7 | M | eBL | Ileum | - | n.a. | + | - | + (*IGL*)  - (*IGK*) | - | + |
| **83** | 8 | M | eBL | Soft tissue | + | + | + | + | n.p. | - | - |
| **84** | 9 | F | eBL | Maxilla | + | n.a. | + | + | n.p. | - | + |
| **85** | 11 | M | eBL | Oral cavity | + | n.a. | + | + | n.p. | - | + |
| **86** | 4 | F | eBL | Lymph node | + | n.a. | + | + | n.p. | - | + |
| **87** | 10 | M | eBL | Maxilla | + | n.a. | + | - | + (*IGL*)  - (*IGK*) | - | + |
| **88** | 7 | M | eBL | Abdomen | - | + | + | n.p. | n.p. | + | - |
| **89** | 12 | F | eBL | Maxilla | - | + | + | n.p. | n.p. | + | - |
| **90** | 5 | F | sBL | Ileum | - | - | + | n.p. | n.p. | + | - |
| **91** | 45 | M | sBL | Stomach | - | - | + | + | n.p. | - | - |
| **92** | 79 | F | sBL | Vagina | - | - | + | + | n.p. | - | - |

*n.a.: not available; n.p.:not performed; EBER: Epstein-Barr Virus-Encoded Small RNA; B.A.: break a part; eBL: endemic Burkitt lymphoma; sBL: sporadic Burkitt lymphoma, FISH: Fluorescence *in situ* hybridization; HIV:human immunodeficiency virus.
