# Supplementary material for: Molecular switch from MYC to MYCN expression in MYC protein negative Burkitt lymphoma cases
Source: Blood Cancer J. 2019 Nov 20;9(12):91. doi: 10.1038/s41408-019-0252-2 (PMC6868231; doi:10.1038/s41408-019-0252-2)
Supplement: Supplementary file 2 — Supplementary Table 2 [file 41408_2019_252_MOESM2_ESM.docx]

| **MYC-FORWARD** | 5’-AGCGACTCTGAGGAGGAAC-3’ |
| --- | --- |
| **MYC-REVERSE** | 5’-TGTGAGGAGGTTTGCTGTG-3’ |
|  |  |
| **MYC-FORWARD** | 5’-AAAGGCCCCCAAGGTAGTTA-3 |
| **MYC-REVERSE** | 5’-GCACAAGAGTTCCGTAGCTG-3 |
|  |  |
| **MYC-FORWARD** | 5’-AAACACAAACTTGAACAGCTAC-3’ |
| **MYC-REVERSE** | 5’-ATTTGAGGCAGTTTACATTATGG-3’ |
